# Electronic Structure Engineering of Cu–Mn Spinel Oxides via Ni Substitution for Enhanced Electrocatalytic Glucose Oxidation

**DOI:** 10.1021/acs.jpcc.6c03237

**Published:** 2026-07-04

**Authors:** Katarzyna Ostrowska, Bartłomiej Lemieszek, Krystian Lankauf, Iga Szpunar, Alexey Maximenko, Bartłomiej Dec, Piotr Jasiński, Sebastian Molin

**Affiliations:** † Advanced Materials Center, Faculty of Electronics, Telecommunications and Informatics, 49557 Gdańsk University of Technology, ul. G. Narutowicza 11/12, Gdańsk 80-233, Poland; ‡ Wallenberg Initiative Materials Science for Sustainability, Department of Chemistry and Chemical Engineering, Chalmers University of Technology, Gothenburg 41296, Sweden; § National Synchrotron Radiation Centre SOLARIS, 37799 Jagiellonian University, Czerwone Maki 98, Kraków 30-392, Poland; ∥ Department of Optoelectronics, Faculty of Electronics, Telecommunications and Informatics, Gdańsk University of Technology, 11/12 Gabriela Narutowicza Street, Gdańsk 80-233, Poland

## Abstract

Diabetes is one of the major civilization diseases of the 21st century. Effective glucose monitoring is a crucial diagnostic method for its active prevention. In addition to widely used enzyme-based sensors, nonenzymatic sensing solutions are being actively developed. The highest sensing performance has been reported for cobalt-containing compounds (i.e., Co_3_O_4_), but certain challenges related to availability and cost necessitate the search for alternative materials. The paper presents the electrocatalytic performance of nickel-doped manganese–copper spinel oxides for glucose detection in aqueous solutions. The spinel oxides were doped with nickel in various mass ratios: CuMn_2_O_4_, CuMn_2‑*x*_Ni­(_*x* = 0.3,0.6,0.9_)­O_4_. Structural analysis was performed to determine their crystalline properties, and the influence of the nickel content on the electrocatalytic activity was systematically investigated. It was found that the amount of nickel significantly affected the sensing properties of the synthesized powders. The most active samples exhibited a sensitivity of approximately 695 μA mM^–1^ cm^–2^ in the linearity range of 0.02–0.1 mM and 406 μA mM^–1^ cm^–2^ in the range of 0.2–1 mM. Electrochemical measurements were further extended to determine selectivity for commonly occurring interfering compounds. Additionally, *operando* X-ray absorption spectroscopy was used to monitor changes in the oxidation states of electrochemically active elements during the glucose oxidation reaction. Theoretical density of states confirmed that nickel doping enhances glucose adsorption and improves the material’s catalytic properties. This work revealed that the Mn^3+^/Mn^4+^ redox pair acts as the dominant electroactive center during glucose oxidation, whereas nickel primarily functions as an electronic structure modulator, stabilizing Mn^4+^-rich surface species. The combined *operando* and theoretical analyses show how Ni addition influences glucose adsorption, charge transfer, and electrocatalytic glucose oxidation in Mn-based spinel oxides.

## Graphical abstract

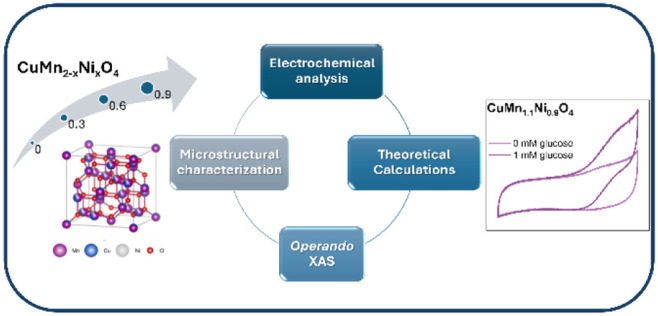

## 1 Introduction

Diabetes and its treatment affect an increasing number of patients worldwide.^1,2^ It is defined as a set of metabolic diseases characterized by hyperglycemia resulting from the incorrect action of insulin and/or defects in its production.^3^ If left untreated, it may lead to multiorgan damage. The key to ensuring successful treatment and maintaining patients’ quality of life is the development of highly effective methods for diagnosing diabetes and monitoring glucose levels.^4,5^

Technologies for glucose sensors are currently divided into two basic solutions: enzymatic (3rd generation) and nonenzymatic sensors (4th generation).^6^ In the case of enzymatic ones, oxidoreductases are used, i.e., enzymes that oxidize glucose to gluconolactone, with glucose oxidase being the most widely used. When determining the amount of glucose, the quantity of hydrogen peroxide produced is measured, as it is directly proportional to the amount of glucose present.^7^

In nonenzymatic glucose sensors, the electrocatalytic oxidation process strongly depends on the electronic structure of the electrode material, surface redox chemistry, adsorption energetics, and charge-transfer kinetics occurring at the electrode/electrolyte interface. Therefore, understanding the relationship among crystal structure, electronic properties, and catalytic activity is crucial for the rational design of advanced sensing materials.

Enzymatic sensors exhibit several significant drawbacks, encompassing issues such as enzyme instability, complexity, and higher production costs.^8^ To reduce these disadvantages, efforts have been directed toward the development of enzyme-free glucose detection methods. As a result, the search for nonenzymatic electrodes as catalysts for the glucose oxidation reaction has intensified. This category of sensors covers a broad spectrum that has been extensively investigated and has demonstrated notable efficiency.^9^ Their primary advantages lie in stability, simplicity, and reproducibility, notably being devoid of impediments associated with a lack of oxygen interference.^10^

Examples of nonenzymatic catalysts for glucose oxidation include platinum,^11,12^ silver,^13,14^ gold,^15,16^ metal oxides,^17,18^ and graphene.^19–22^ From the variety of different materials, spinel oxides, characterized by the general AB_2_O_4_ formula (where A and B represent metal ions), have garnered attention due to the expansive array of elements capable of occupying positions within the crystal structure.^23,24^ Not only do these oxides exhibit notable electrocatalytic properties,^25,26^ but they also hold promise as potential glucose sensors.^27,28^

Diverse compositions were tested for this specific purpose, encompassing cobalt^29^ and iron^30^ spinel oxides doped with additional elements such as nickel,^27,28,31^ manganese,^32^ or copper.^33^ However, due to the limited availability and fluctuating cobalt prices, alternative catalyst materials are being sought.^34,35^ Cobalt is included on the European Union’s Critical Raw Materials (CRM) list due to its strategic importance for energy storage technologies and the high risk of supply chain disruptions. The global supply of cobalt is highly geographically concentrated and burdened with significant environmental and social issues, which increases its criticality as a raw material.^36^ Copper and manganese spinel oxides can be considered viable alternative electrode materials,^37^ though they have been studied to a much lesser extent.

Previous studies on Ni-doped spinel oxides have mainly focused on OER/ORR electrocatalysis, where Ni incorporation was associated with conductivity enhancement and optimization of oxygen intermediate adsorption. However, the mechanistic role of Ni doping in glucose electrooxidation processes remains insufficiently understood, particularly in Mn-based spinel systems.^38–42^

Moreover, despite reports describing improved electrochemical performance after Ni incorporation, the majority of previous studies relied mainly on ex situ characterization techniques and did not directly investigate the evolution of electroactive centers under operating glucose oxidation conditions. Consequently, the relationship among Ni-doped electronic structure modification, surface redox reconstruction, and glucose oxidation kinetics remains unclear.

In this study, we tested the manganese–copper spinel oxides substituted with nickel (CuMn_2‑*x*_Ni­(_*x* = 0, 0.3, 0.6, 0.9_)­O_4_) as materials for catalyzing glucose oxidation reactions. The investigation aimed to determine whether an increase in the amount of doped nickel has a positive influence on the efficiency of glucose sensing and to explore its impact on the structural attributes of these materials. This paper comprehensively describes the synthesis and preparation procedures of the materials, including an in-depth examination of their physical, morphological, and electrochemical characteristics. Additionally, the study identifies the active sites during operational conditions through *operando* characterization. Theoretical calculations were also performed to confirm the effect of nickel doping on the electrocatalytic properties.

Importantly, the present work provides mechanistic insight beyond the conventional interpretation of Ni doping in spinel oxides reported for OER/ORR electrocatalysis and energy-storage applications. This study demonstrates that Ni doping in Cu–Mn spinel oxides fundamentally modifies the glucose electrooxidation pathway through electronic structure engineering and Mn valence redistribution.

By combining *operando* XAS/EXAFS analysis, electrochemical measurements, and theoretical calculations, this work reveals that the Mn^3+^/Mn^4+^ redox pair acts as the dominant electroactive center during glucose oxidation, whereas Ni primarily functions as an electronic structure modulator, stabilizing Mn^4+^ species. Furthermore, calculations demonstrate that Ni incorporation significantly enhances glucose adsorption energetics and promotes stronger molecule–surface interactions through modification of the orbital occupancy. These findings demonstrate that nickel doping not only improves the electronic and catalytic properties of the spinel oxide but also enhances glucose adsorption and charge-transfer processes, leading to more efficient nonenzymatic glucose oxidation. The presented results provide a deeper understanding of the role of Ni doping in Mn-based spinel oxides and may support the future design of high-performance electrochemical glucose sensors.

## 2 Methods

### 2.1 Powder Preparation

The powders were synthesized using the sol–gel method.^43^ Appropriate quantities of selected nitrates, Cu­(NO_3_)_2_·3H_2_O, Mn­(NO_3_)_2_·4H_2_O, and Ni­(NO_3_)_2_·6H_2_O (99.9% purity, Sigma-Aldrich), were dissolved in a minimal volume of deionized water. A similar procedure was applied to citric acid, followed by the addition of ethylenediaminetetraacetic acid (EDTA) and ammonia solution. The mixture was stirred until fully dissolved over a 10 min period using a magnetic stirrer. Additional amounts of ammonia solution (25% solution, POCH, Poland) were added to maintain the pH at 6, considered the optimal value for crystal structure formation.^44^ The pH of the solution was monitored using pH indicator paper. The quantities of reagents used followed the specific molar ratio: TMI:CA:EDTA = 1:2:1 (TMI = Total Metal Ions).

Subsequently, the solution was heated, while being stirred with a magnetic stirrer, to 90 °C for 5 to 7 h, to facilitate the maximum evaporation of the ammonia solution and form a dense gel. The prepared gel was then dried in a laboratory oven at 95 °C for 5 h, 120 °C for 4 h, and 180 °C for 2 h. The product was then ground into a fine powder using an agate mortar, placed in an alumina crucible, and calcined at 800 °C for 2 h in a muffle furnace. After thermal treatment, the powders were further ground and pressed into pellets, which were annealed by heating to 800 °C for 2 h and then rapidly quenched to room temperature to stabilize the structure.^45^

The pellets were subsequently transformed back into a powdered state by using an agate mortar. To improve the electrocatalytic surface area, the powders were milled by using spherical grinding media in the form of yttria-stabilized zirconium oxide (YSZ) balls (1 mm diameter). The powder with isopropanol was placed in 20 mm-diameter glass vials. The vials were horizontally positioned on a roller (Zoz GmbH Rollermill RM1) for 144 h at a rotation speed of 100 rpm.^46^

### 2.2 Microstructural Characterization

Scanning electron microscopy (SEM) (ThermoFisher Phenom XL microscope) was used to analyze the grain size of the powders and to assess the effect of milling. Energy Dispersive X-ray Spectroscopy (EDS) (ThermoFisher Escalab 250Xi, source gun type Al Kα with a 500 μm spot size at a pass energy of 150 eV) was performed to confirm the Mn:Ni ratio. To visualize the crystallographic structure and to present the influence of nickel addition on the samples’ crystalline properties, powder X-ray diffraction (pXRD) was performed using a Bruker D2 Phaser diffractometer with CuKα radiation (λ = 1.5404 Å).

To investigate the local geometric and electronic structure, X-ray absorption near-edge spectroscopy (XANES) transmission spectra were acquired at the PEEM/XAS (X-ray absorption spectroscopy) beamlines PIRX and ASTRA of the SOLARIS National Synchrotron Radiation Centre (Cracow, Poland) for the L_3_-edge and K-edge of Mn, Ni, and Cu. These spectra were used to determine the valence states of the metallic elements in the samples. Additionally, reference samples with known valence states of elements were used (MnO (Mn^2+^), Mn_2_O_3_ (Mn^3+^), MnO_2_ (Mn^4+^), NiO (Ni^2+^), CuO (Cu^2+^), and Cu_2_O (Cu^1+^)).

Further *operando* XAS analysis of glucose sensors was conducted under operating conditions. The spectra at the Mn, Ni, and Cu K-edges in fluorescence mode were collected at the ASTRA beamline at the SOLARIS National Synchrotron Radiation Centre in Cracow, Poland. *Operando* XAS offers significant insights into the nature of the catalytically active sites, providing a more accurate understanding of the electronic processes occurring in the system during the glucose oxidation reaction. For the *operando* studies, a cell was designed and 3D-printed (Figure S2). Because of the water-absorbing properties, the measurements were performed in fluorescence mode. A portion of electrocatalytic spinel was deposited on conductive carbon paper. Spinel was applied in the form of the same ink as described, but with a significantly increased solid (catalyst and CCB) concentration (10 times higher). The concentration was increased in order to obtain the strongest possible signal in the detector. To connect the carbon paper with the potentiostat, a PTFE holder with a Pt connection was used. A window of the cell was secured with Kapton tape, and then a carbon paper working electrode was attached to it (also using Kapton tape). We wanted to get maximum contact of the electrode with the surface of the tape on the window, in order to minimize the effect of radiation absorption by water. As before, data were collected in a 3-electrode measurement system. To collect the electrochemical signal, the IVIUM Technologies Vertex 5A potentiostat was used. Mn, Ni, and Cu K-edges were measured during chronoamperometry at 0.5 V vs Ag/AgCl without and with the addition of glucose (0 and 1 mM concentration, respectively). Before the actual measurements were started, a spectrum of the measurement chamber in which the cell was placed was collected to eliminate the background signal. The cell was placed at an appropriate angle in the chamber to reflect the signal directly to the detector. In order to compare the results obtained for each element, reference compounds with known oxidation states were also measured.

### 2.3 Fabrication of Modified Electrode

A rotating disc glassy carbon electrode (RDE-GCE, 0.196 cm^2^, ALS Co., Ltd.) was used as the working electrode. As a pretreatment, the RDE-GCE electrode was polished on an automatic polisher using a diamond solution (1 μm). Following this procedure, the electrode was sonicated in isopropanol and deionized water for 10 min and then air-dried for 24 h.

To apply the electrocatalytic material onto the RDE-GCE electrode, an ink with spinel powder, Conductive Carbon Black (CCB), Nafion 117 solution (Sigma-Aldrich), and isopropanol was prepared. The CCB was previously ball-milled using 1 mm YSZ balls in isopropanol for 144 h, using the same procedure as for the catalyst powder.^46^ To generate 0.5 mL of ink, catalyst:CCB:Nafion were added in a mass ratio of 5:5:2 and filled up with 500 μL of isopropanol. The ink was ultrasonicated in an ice–water bath for 30 min.

After ink preparation, while the RDE-GCE electrode was rotated at 500 rpm, 5 μL of the ink suspension was drop-cast directly onto the glassy carbon surface, achieving a catalyst mass loading of 45.5 μg. The rotation helped to get a uniform surface without aggregates. A mask cut from tape was prepared to concentrate the ink on the GCE core area (ø 5 mm). After ink application, the electrode was dried for a minimum of 12 h in a desiccator at room temperature to evaporate the solvent from the ink. An example of the prepared modified electrode is presented in Figure S1 in the SI.

### 2.4 Electrochemical Measurements

Electrochemical measurements were conducted by using a BioLogic BP-300 station with a three-electrode system. The RDE-GCE was modified with the electrocatalyst, and used as the working electrode , and an Ag/AgCl (sat. KCl) electrode (ALS Co., Ltd., Japan) and a Pt coil were used as the reference electrode (RE) and counter electrode (CE), respectively.

All measurements were performed in a 0.2 M KOH solution prepared from Merck’s 1 M KOH Titripur. In the design of glucose nonenzyme sensors, the supporting electrolyte is usually an alkaline solution with a concentration of 0.1–0.5 M. Due to the low glucose activity under acidic conditions, there are few reports about it.^47^ During the experiments, the RDE rotated at 1600 rpm to ensure uniform dispersion of the injected glucose in the solution and to perform proper signal acquisition.

CV measurements were conducted in the range from 0 to 0.6 V vs RE at a scan rate of 100 mV s^–1^ for each sample, both in the absence of glucose and at a concentration of 1 mM glucose. Based on the CV curves, a potential of 0.5 V vs Ag/AgCl (sat. KCl) was selected, as it exhibited clear glucose oxidation without a significantly high oxygen evolution reaction current.

Chronoamperometry (CA) measurements were performed at this constant potential for 200 s to allow stabilization of the oxidation process at the electrode surface. The final stable 50 s of each CA response were selected for analysis. CA responses were recorded for increasing concentrations of glucose in the solution (0, 0.02, 0.04, 0.06, 0.08, 0.1, 0.2, 0.4, 0.6, 0.8, and 1 mM). Between CV and CA measurements, the electrolyte was replaced with a fresh, clean solution. At least three samples were prepared and measured for each powder.

### 2.5 Theoretical Calculations

All calculations were performed using the QuantumATK package (version V-2023.12, Synopsys)^48^ employing the linear combination of atomic orbitals (LCAO) method and norm-conserving PseudoDojo pseudopotentials.^49^ The electronic structures of the studied systems were computed using the Heyd–Scuseria–Ernzerhof (HSE06) hybrid functional.^50^ In the DFT calculations, the Brillouin zone was sampled by a Monkhorst–Pack k-point grid of 4 × 4 × 4 points. A density mesh cutoff of 100 hartree and a broadening of 1000 K were selected.

The DOS calculations were performed on a denser 7 × 7 × 7 k-point grid using the tetrahedron method. Molecular dynamics (MD) simulations using machine-learned potentials (MACE L2 model)^51^ were carried out within QuantumATK using the TremoloX calculator.^52^ In addition, dispersion interactions were taken into account by performing the simulations using the DFT-D3 correction method^53^ with Becke–Johnson damping, accounting for the dispersion forces between the glucose, the surface, and the water molecules.

Adsorption calculations were performed on a single 3 × 3 (316 Å^2^) CuMn_*x*_Ni_*x*_O_4_ surface using a number of glucose molecules ranging from 1 to 9. The electronic properties were calculated on a bulk structure consisting of 12 atoms, belonging to *R*3̅*m* space group and D_3_d^5^ symmetry group. The initial bulk structure, with a volume of 146 Å^2^, was relaxed to 148.34 Å^2^ (undoped) and 143.69 Å^2^ (with Ni dopant).

## 3 Results

### 3.1 Material Characterization

Powder X-ray diffraction method was used to determine the crystal structure of the investigated materials. The collected XRD patterns of the as-prepared and ball-milled Ni-substituted CuMn_2_O_4_ powders are shown in Figure 1a and b, respectively. Phase analysis revealed a spinel structure, with most of the peaks indexed based on the crystallography data for CuMn_2_O_4_,^54^ corresponding to the *Fd*3̅*m* space group (No. 227). However, in all of the investigated materials, additional peaks were observed, which were assigned to the presence of CuO. The peak intensity increases with the increasing nickel content, suggesting that the strain induced by the difference in manganese and nickel ionic radii is released with the CuO precipitation. In the sample with the highest nickel content, the presence of nickel oxide was observed, which suggests that the solubility limit was exceeded. In the undoped material, the presence of manganese oxide was revealed. Significant peak broadening was observed in the materials after milling due to the decrease in material crystallinity and grain size. After milling, the presence of secondary phases is less pronounced, but it is rather related to peaks overlapping, not phase dissolution. In all of the investigated materials, Ni tends to substitute manganese in the octahedral coordination, which has also been previously observed in Ni-substituted manganese spinel oxides.^55^

**Figure 1:**
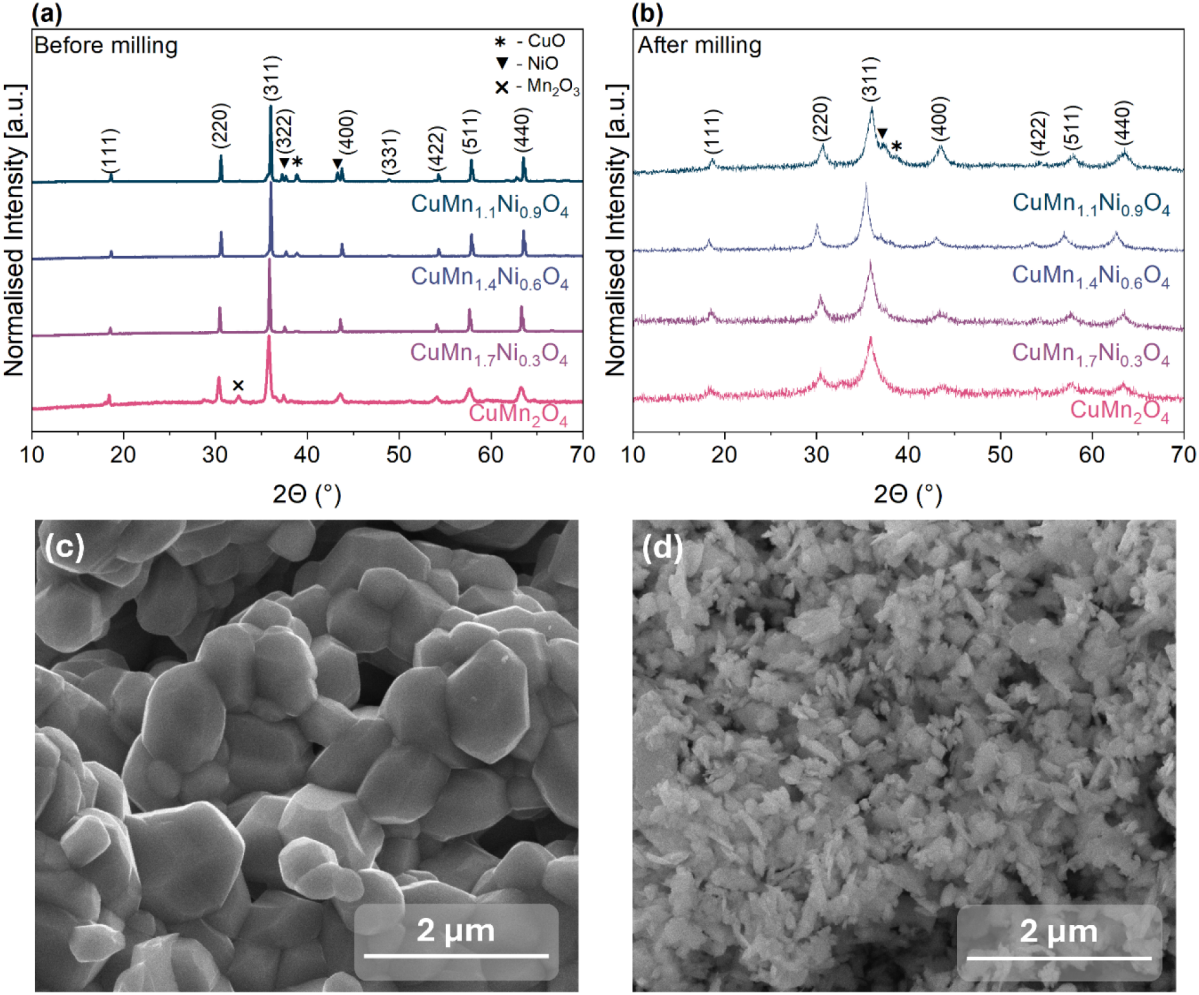
XRD patterns of Ni-substituted samples: (a) before and (b) after milling. SEM image (×10000) of (c) as-synthesized and (d) ball-milled CuMn_1.1_Ni_0.9_O_4_ powder.

Powder morphology was characterized on the as-synthesized as well as on the ball-milled powder. The milling process was carried out to increase the electrochemically active surface area, yielding favorable electrochemical performance.^46^ Figure 1c-d presents the effect of the particle size reduction induced by the milling procedure. The average grain size diameter before milling was ∼1 μm and was reduced by an order of magnitude to ∼100 nm, which is in strong agreement with the XRD data. Ball milling causes a 10-fold reduction in grain size, which leads to higher active sites and more space for reactions to occur. The addition of nickel and the presence of secondary oxides do not significantly affect the mechanical properties, since there is no relation between Ni content and grain size after milling. SEM images of other prepared powders after milling are presented in Figure S3 in the SI. The EDS results, presented in Table S2, confirmed the Mn:Ni ratio of the prepared powders.

Analysis of XANES spectra determined the effect of nickel addition on the valence states of other elements. The Mn L_3_-edge analysis revealed the most significant changes (Figure 2a). The spectrum of the manganese–copper spinel overlaps with the MnO and Mn_2_O_3_ phases (peaks at energy ∼640.25 eV for Mn^2+^, ∼641.62 and ∼642.18 eV for Mn^3+^). However, the nickel-substituted samples exhibit shifts correlated with the change of the oxidation state of Mn cations toward +4. This is indicated by decreasing the signal intensity for energy ∼640.91 eV and the shift of the measured signal toward energy ∼643.1 eV, what is characteristic for Mn^4+^. We observe lower intensity of the peaks characteristic for the +2 and +3 oxidation states with a clear increase in the peaks coming from the MnO_2_ phase. The addition of Ni^2+^ cations thus exerts an influence on the manganese valency. The spectral changes are noticeable even at the smallest amount of doped nickel.

**Figure 2:**
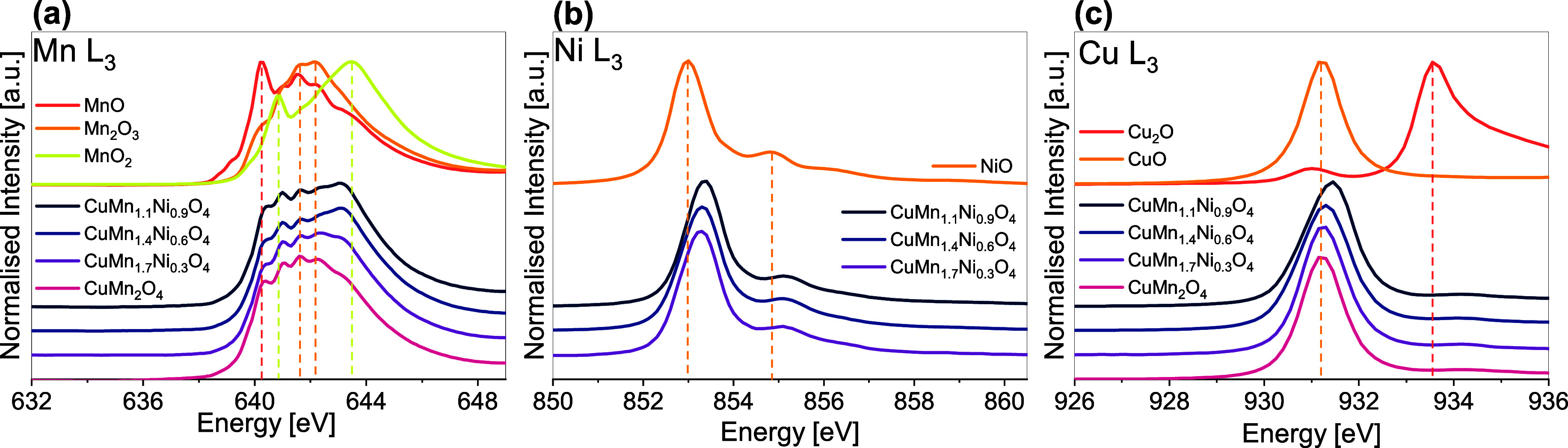
XANES L_3_-edge spectra of (a) Mn, (b) Ni, and (c) Cu.

The results obtained for L_3_-edge of nickel indicate that it occurs in the +2 oxidation state (Figure 2b). This is indicated by the increases in intensity at energies ∼852.99 and 854.84 eV, which correspond to the NiO signal maxima. However, a gradual shift of the signal toward higher energies is visible, which may indicate a slight increase in the oxidation state of nickel with an increase in its amount in the spinel structure.

Regarding the copper L_3_-edge (Figure 2c), the signal peaks’ maxima notably align with the reference spectrum of CuO (energy of ∼931.22 eV), suggesting that copper exists in the +2 oxidation state in the tested spinel oxides. However, similarly to nickel, a gradual shift of the peaks toward higher energies is noticeable, which may indicate a slight decrease in the oxidation state of copper.

To additionally confirm the increase in the manganese oxidation state, K-edge XANES spectra were measured. Figure 3 presents the spectra for the Mn K-edge for every prepared compound and references with known oxidation states of manganese (MnO (Mn^2+^), Mn_2_O_3_ (Mn^3+^), and MnO_2_ (Mn^4+^)). To create the calibration curve, the mid-energy of the edge was considered (energy in the middle of the edge). Based on the calibration curve (Figure 3 c) and linear fitting, we can calculate the Mn oxidation state of CuMn_2_O_4_, CuMn_1.7_Ni_0.3_O_4_, CuMn_1.4_Ni_0.6_O_4_, CuMn_1.1_Ni_0.9_O_4_ as ∼+3.08, +3.33, +3.59, and +3.57, respectively. It can be stated that in the given compounds, manganese occurs between +3 and +4 oxidation states, and with increasing nickel content, the +4 state becomes more dominant. A slight deviation from the trend in the sample with the highest nickel content may be caused by the presence of additional phases (NiO and CuO), whose occurrence was confirmed by XRD results.

**Figure 3:**
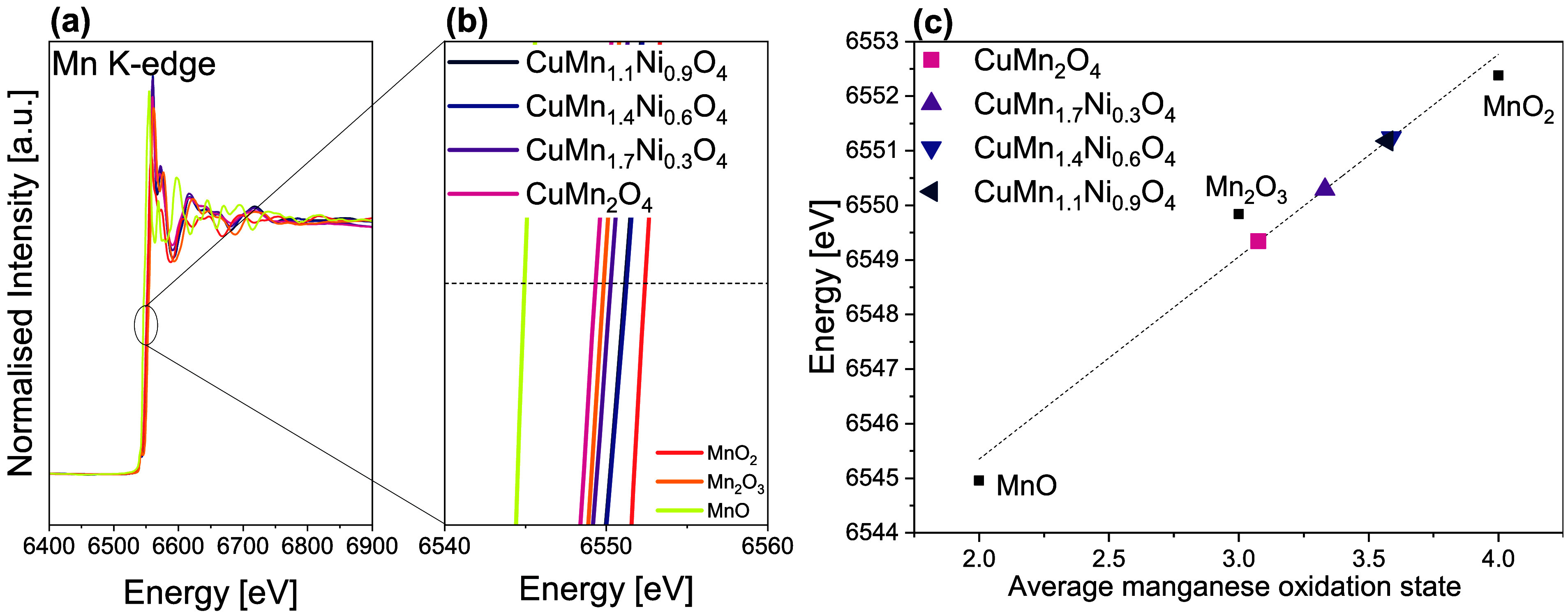
XANES K-edge spectra of (a-b) Mn and (c) the average manganese oxidation state calculated from mid-energy.

Predominantly, Mn^2+^ cations occupy tetrahedral sites (A) within the crystal lattice of manganese–copper spinel, while Cu^2+^, Mn^3+^, and Mn^4+^ are situated in octahedral sites (B).^56^ Consequently, nickel doping leads to an increase in the number of manganese cations in the octahedral sites. This increase in the presence of Mn^3+/4+^ in the octahedral sites contributes to enhanced electrical conductivity^57^ and a rise in the number of active sites, thereby influencing the electrocatalytic efficiency,^58,59^ which should have an effect on the electrochemical activity of the material.

### 3.2 Electrochemical Analysis

The change in the electrochemical response of the catalyst upon the addition of glucose and the gradual increase in its concentration in the solution were investigated. First, CV curves were recorded without glucose and after its addition (Figure 4a). Examination of the CA recordings for each reported concentration (Figure 4b) shows that the current response is visible even at the lowest concentrations of added glucose (0–0.1 mM in 0.02 mM increments). A comparison of the measurement responses for 1 mM glucose across different compounds is presented in Figure 4c. The highest current was observed for the CuMn_1.1_Ni_0.9_O_4_ sample, i.e., for the highest Ni content. An electrochemical response to glucose was evident for each of the discussed compounds.

**Figure 4:**
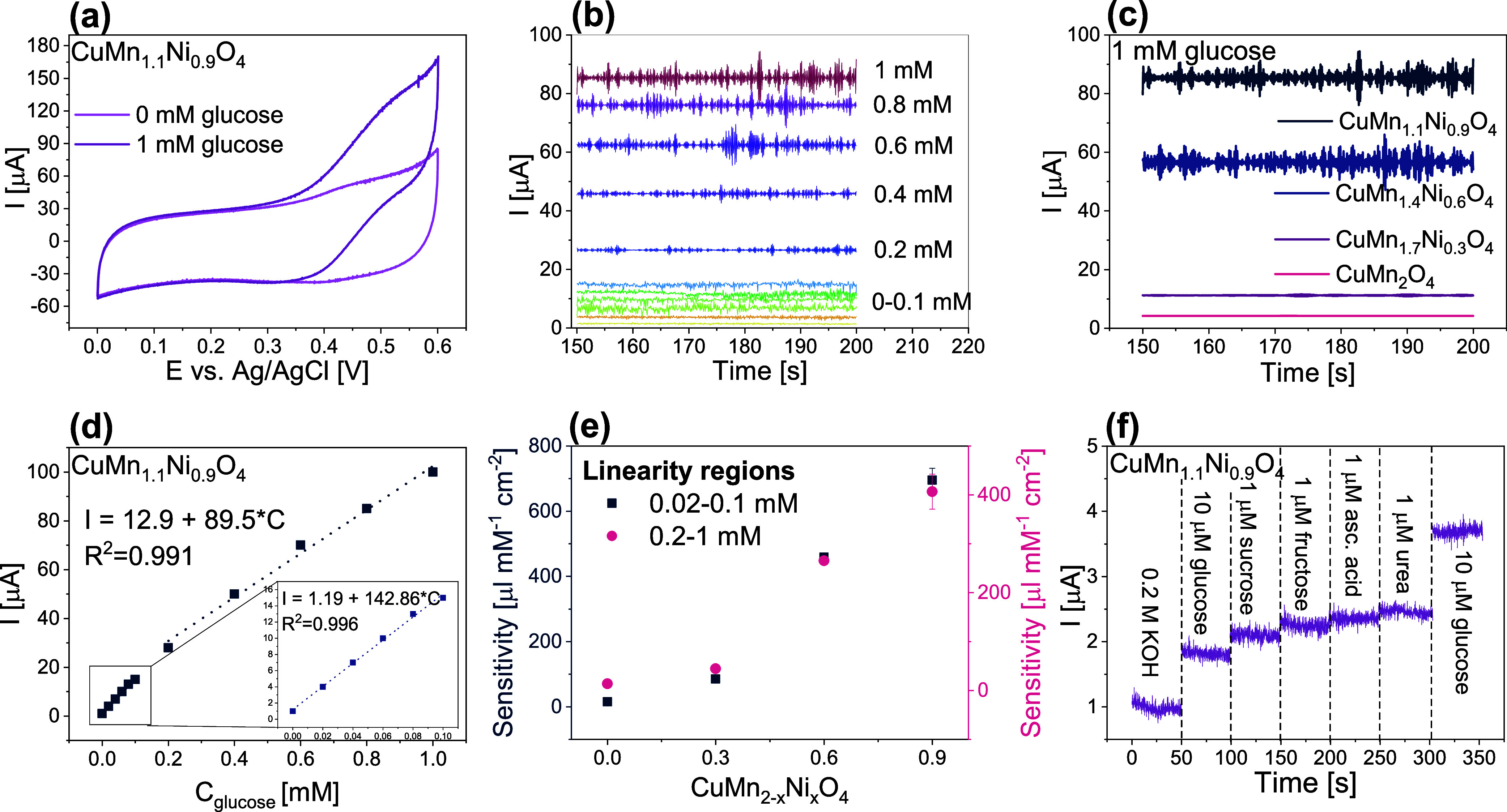
(a) CV curves in the absence and presence of 1 mM glucose for CuMn_1.1_Ni_0.9_O_4_, (b) CA response for CuMn_1.1_Ni_0.9_O_4_ sample from 0 to 1 mM of glucose, (c) CA responses for all samples at 1 mM of added glucose, (d) an example of a calibration curve with two linearity regions, (e) comparison of sensitivity for the two linearity regions with error bars, and (f) selectivity toward selected compounds.

The sensitivity of the electrode is determined by the ratio of the slope of the calibration curve, representing the current–concentration relationship, to the area of the electrode exposed to the electrolyte.^60^ The determined working electrode area was 0.196 cm^2^. On prepared calibration curves two linearity regions were observed—0–0.1 mM and 0.2–1 mM. For CuMn_2_O_4_, the lower region starts from 0.04 mM. Between 0 and 0.04 mM, we did not observe any increase in the current response for this compound. This indicates lower glucose-sensing properties than those of the samples with nickel doping. An illustrative curve is presented in Figure 4d. Consequently, the sensitivity for CuMn_2‑x_Ni_*x*_O_4_ (*x* = 0, 0.3, 0.6, 0.9) was calculated as follows: for the lower region—15, 85, 458, 695 μA mM^–1^ cm^–2^, and for higher region—13, 44, 265, 406 μA mM^–1^ cm^–2^. Figure 4 e shows a comparison of the sensitivity in the two linearity regions. A noticeable positive influence of nickel substitution on the current response was observed. Calibration curves for samples with lower nickel content are presented in Figure S4.

As part of the selectivity analysis, the CuMn_1.1_Ni_0.9_O_4_ sample was selected, and CA measurements were conducted with the gradual addition of other potentially active compounds: sucrose, fructose, ascorbic acid, and urea (Figure 4f). These compounds were chosen due to their common presence in human body fluids.^61–63^ The interfering substances were added as solutions to get a final concentration of 1 μM in the electrolyte. A clear increase in the current response is evident upon the addition of glucose, showing more than a twofold increase in current. A slight increase in activity was noted with the addition of sucrose and fructose, possibly due to their structural similarity to glucose. However, the further addition of glucose resulted in a significantly increased current signal compared to the interfering media. The addition of ascorbic acid and urea does not appear to affect the current response. Therefore, it can be concluded that the tested materials do not exhibit a significantly increased current response to common organic interferents, and this proves the selectivity properties of the material.

In Table 1, the summary of literature results on the glucose detection performance of spinel oxides is shown. The discussed compounds show higher or comparable electrocatalytic activity toward the glucose oxidation reaction in a similar linearity range to those presented in the literature, and the data can be discussed in relation to the existing state of the art. In comparison to manganese–cobalt spinel oxides presented in previous work,^64^ those shown in this study demonstrate a notable increase in sensitivity toward glucose detection. Specifically, the CuMn_1.1_Ni_0.9_O_4_ sample shows a notable increase in sensitivity compared to MnLi_0.1_Co_1.9_O_4_ (73.1 μAm M^–1^cm^–2^). For further comparison, the CuMn_2_O_4_ spinel reported by Cui et al., prepared using the hydrothermal-calcination method on porous Ni foam and tested in the glucose concentration range of 0.15–1.7 mM, exhibited a sensitivity of 12.5 μAm M^–1^cm^–2^.^60^ This is a lower value compared to the discussed CuMn_2_O_4_, which demonstrated a sensitivity of 17 μA mM^–1^cm^–2^.

**Table 1:** Summary of Literature Results Concerning the Performance of Glucose Electrodes Based on Spinel Oxides

Electrode material	Working potential [V]	Sensitivity [μA mM^–1^ cm^–2^]	Linear range	Ref
CuMn_2_O_4_ SS/NF	+0.4	12.520	0.15–1.7 mM	^60^
Electrospun Co_3_O_4_ nanofibers	+0.59	36.25	Up to 2.04 mM	^65^
Mesoporous NiCo_2_O_4_ nanowires/GCE	+0.44	72.4	0.37–2000 μM	^66^
Graphene-wrapped porous Co_3_O_4_/NiCo_2_O_4_ double-shelled nanocages/GCE	+0.5	304	10–3520 μM	^67^
APBA@CoFe_2_O_4_@N-CNFs	+0.55	318	0.01–3.52 mM	^30^
MnCo_2_O_4_ nanofibers/GCE	+0.55	679.5	0.05–800 μM	^68^
MnLi_0.1_Co_1.9_O_4_/GCE	+0.5	73.1	0.02–1 mM	^64^
CuMn_2_O_4_/GCE	+0.5	*15*	0.02–0.1 mM	This work
		13	0.2–1 mM	
CuMn_1.1_Ni_0.9_O_4_/GCE	+0.5	695	0.02–0.1 mM	This work
		406	0.2–1 mM	

### 3.3 *Operando* X-Ray Absorption Spectroscopy

*Operando* XAS is a useful technique for revealing the electronic and geometric structure of electrocatalysts under operational conditions.^69,70^ This paper presents results from the *operando* XAS investigation of nickel-substituted manganese–copper oxide spinels during glucose addition. The primary objective of this experiment was to gain insight into structural and electronic state changes of Ni, Cu, and Mn. Combining *operando* XAS with standard electrochemical measurements enables the identification of the actual active sites involved in the glucose oxidation reaction in the presence of a catalyst, thereby supporting further materials development. The sample with the best electrocatalytic properties for glucose detection, i.e., CuMn_1.1_Ni_0.9_O_4_, was selected for analysis, as well as a sample without nickel doping for comparison. Figure 5 shows k-space oscillation plots and Fourier transform (FT) (k^2^ χ­(k)) plots (without phase correction) of *operando* EXAFS results.

**Figure 5:**
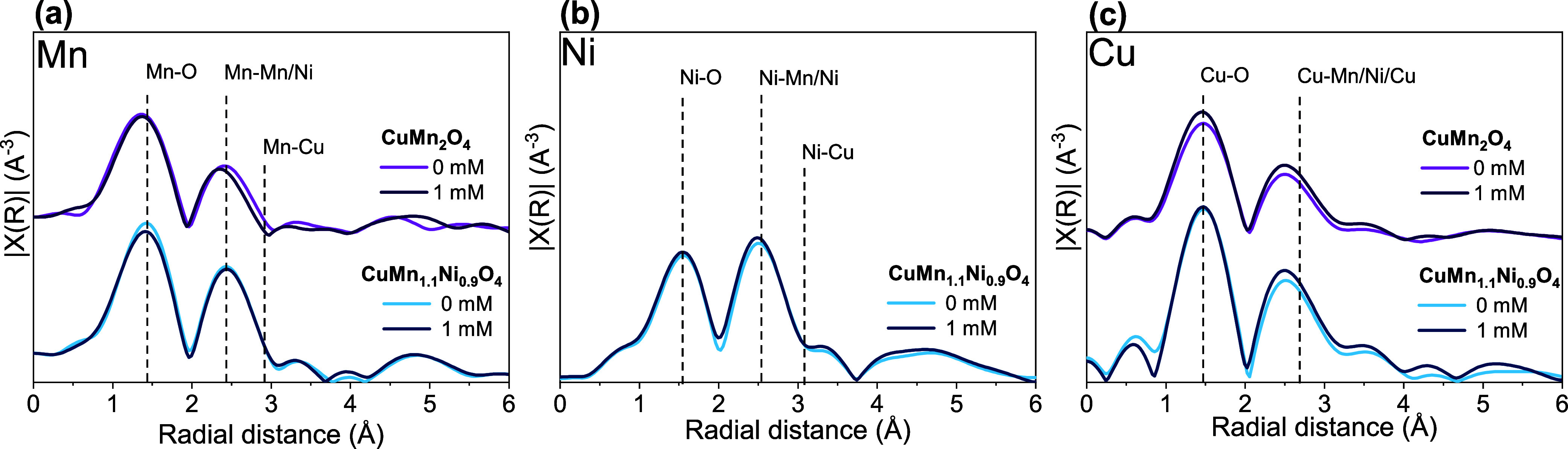
R-space Fourier transform (k^2^ χ­(k)) EXAFS for CuMn_2_O_4_ and CuMn_1.1_Ni_0.9_O_4_ at (a) Mn, (b) Ni, and (c) Cu K-edge.

In the case of manganese (Figure 5a), for the CuMn_2_O_4_ sample, the peak at a radial distance of ∼1.3 Å is associated with the Mn–O bond. For the undoped sample, after adding glucose to the solution, no change in the peak intensity is observed. In contrast, for the nickel-doped sample, a decrease in the intensity of this peak is visible after the addition of glucose. This indicates a change in the amount of oxygen coordination around manganese, which can also be associated with the reduction of the manganese oxidation state.

For Ni, no significant change in the intensity of the peak characteristic of the Ni–O coordination is observed after the addition of glucose (Figure 5b). This may indicate a lack of electrochemical activity of nickel in this compound during the glucose oxidation reaction.

In the case of copper (Figure 5c), an increase in the signal intensity in the Cu–O region (∼1.46 Å) is observed after the addition of glucose for the CuMn_2_O_4_ sample. For the Ni-doped sample, no change in the signal intensity in the region is observed before and after glucose addition.

By analyzing the behavior of these elements in their oxygen coordination environments before and after glucose addition, it can be concluded that nickel incorporation induces the reduction of manganese during the electrochemical glucose oxidation reaction. The presence of nickel also affects the behavior of copper—the absence of changes observed for the Ni-doped sample after glucose addition suggests that copper becomes less electrochemically active. Measured *operando* XANES K-edge spectra are presented in Figure S5. The determined vibration spectra (Figure S6) and Fourier transform fit values for the best fit (Table S2) are presented in SI.

### 3.4 Theoretical Calculations

The density of states (DOS) shown in Figure 6a reveals significant modifications in the electronic structure upon nickel doping in CuMn_2_O_4_. Two key observations emerge from the comparison of pristine CuMn_2_O_4_ (blue curve) and Ni-doped CuMn_1.5_Ni_0.5_O_4_ (green curve). The Fermi level for CuMn_2_O_4_ is located at −7.10 eV, whereas for CuMn_1.5_Ni_0.5_O_4_, it shifts to a higher energy of −7.64 eV. This upward shift suggests an increase in the availability of electrons for redox processes, potentially enhancing the material’s catalytic activity and electrochemical performance. Despite the Fermi level shift, the Ni-doped material exhibits a larger band gap near the Fermi level compared to the pristine compound. This suggests that while electrons are more energetically accessible, transitions across the band gap may require higher activation energy, potentially influencing charge transport properties. Figure 6b and c shows the crystal structures of CuMn_2_O_4_ and CuMn_1.5_Ni_0.5_O_4_, respectively. In the nondoped structure (b), the bond lengths are Mn–O: 1.94 Å and Cu–O: 1.98 Å, indicating a relatively uniform bonding environment. In the doped structure (c), nickel atoms (green) partially replace manganese, leading to changes in bond lengths: Mn–O decreases slightly to 1.91 Å, Cu–O increases to 2.01 Å, and Ni–O forms longer bonds at 2.04 Å.

**Figure 6:**
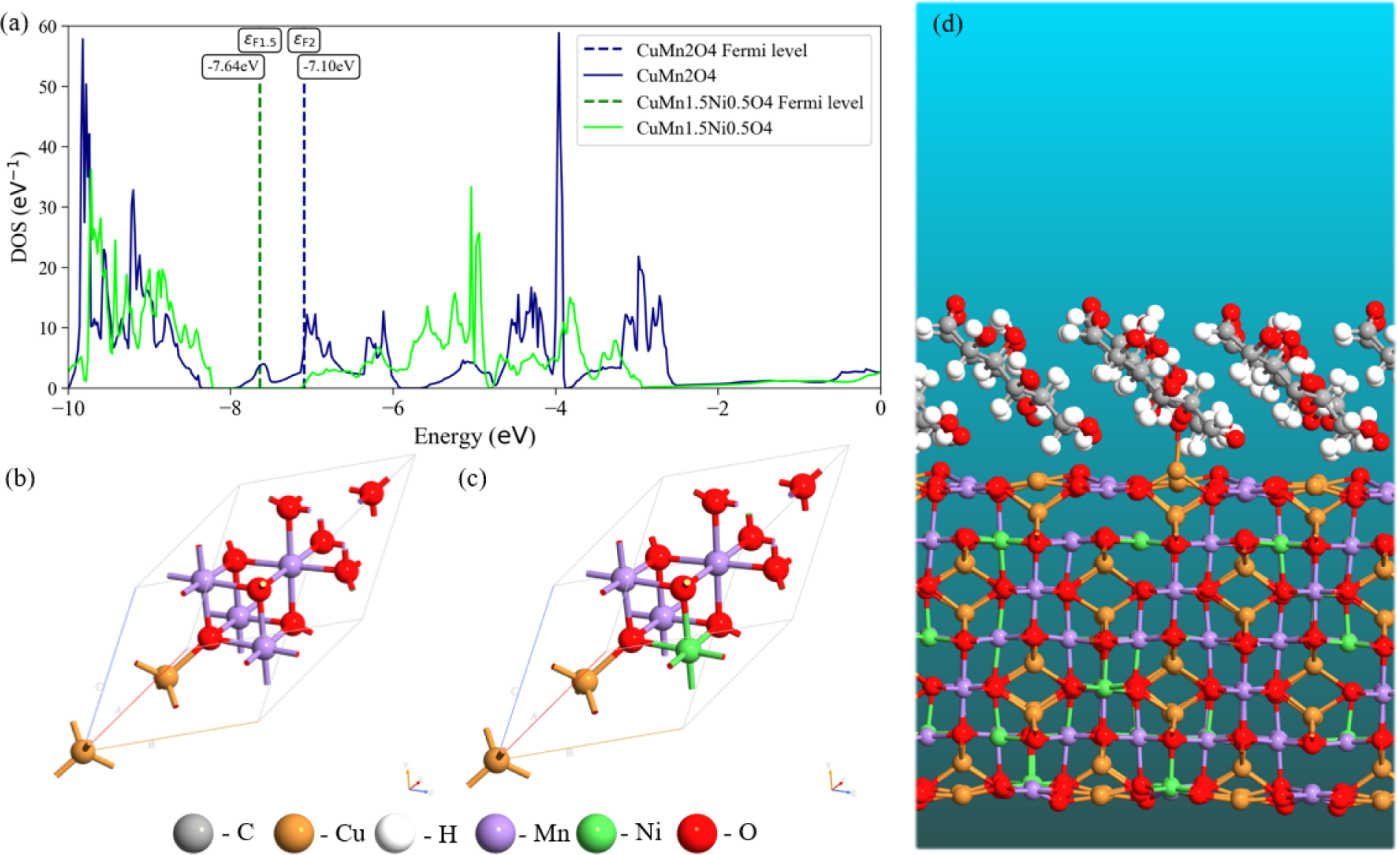
(a) Density of states (DOS) for CuMn_2_O_4_ (blue curve) and CuMn_1.5_Ni_0.5_O_4_ (green curve). The Fermi levels are marked with dashed vertical lines: −7.10 eV for CuMn_2_O_4_ and −7.64 eV for CuMn_1.5_Ni_0.5_O_4_. The DOS of the Ni-doped material exhibits a shift in the Fermi level to higher energies and an increased band gap around the Fermi level, indicating significant changes in the electronic structure due to nickel substitution. (b) Crystal structure of CuMn_2_O_4_, showing the arrangement of Cu (orange), Mn (purple), and O (red) atoms. (c) Crystal structure of CuMn_1.5_Ni_0.5_O_4_, highlighting the substitution of Mn with Ni atoms (green). These structural and electronic modifications influence the material’s redox properties and catalytic performance. (d) The panel shows the surface of CuMn_1.5_Ni_0.5_O_4_ in the presence of glucose molecules in water. The visualization highlights the adsorption behavior and orientation of glucose on the doped spinel surface.

As shown in Figure 6d, the surface of CuMn_1.5_Ni_0.5_O_4_ exhibits no notable changes in the glucose adsorption mechanism when compared to nondoped surfaces. Nevertheless, simulation results indicate that nickel doping reduces the glucose adsorption density from 35 Å^2^ per molecule to 40 Å^2^ per molecule, while simultaneously increasing the adsorption energy from −2.22 eV to −2.50 eV. Interestingly, at molecule densities of approximately 105 Å^2^ per molecule, glucose molecules exhibit a “laying down” orientation on their side regardless of doping levels, whereas this behavior is not observed at lower molecule densities. Furthermore, adsorption density is strongly dependent on nickel content, while surfaces with compositions, such as CuMn_1.5_Ni_0.5_O_4_, show adsorption densities similar to undoped surfaces, higher doping levels (e.g., CuMn_1.5_Ni_0.5_O_4_ and CuMn_1.25_Ni_0.75_O_4_) result in shifts in adsorption density and increased interaction strength with glucose molecules on the surface.

In the absence of water, nickel doping significantly increases the adsorption density of glucose molecules on CuMn_*x*_Ni_*x*_O_4_ surfaces. For undoped CuMn_2_O_4_, the adsorption density is 63 Å^2^ per molecule, while for CuMn_1.75_Ni_0.25_O_4_ and CuMn_1.5_Ni_0.5_O_4_, it decreases to 40 Å^2^ per molecule and further to 35 Å^2^ per molecule for CuMn_1.25_Ni_0.75_O_4_. Nickel doping increases the surface’s affinity for glucose, and the surface becomes more reactive toward glucose binding. With increasing nickel content, the adsorption energy reaches values as high as 2.64 eV for CuMn_1.25_Ni_0.75_O_4_. Despite an adsorption energy of 2.61 eV for CuMn_2_O_4_, which is comparable to the doped surface, the change in the density values (∼two times higher for CuMn_1.75_Ni_0.25_O_4_) indicates that the change in glucose adsorption density is the primary mechanism driving the change in conductance.

### 3.5 Occupancy of *e*_*g*_/*t*_*2g*_ Orbitals

To understand the possible origin of the increased activity of the nickel-substituted samples, an activity descriptor based on the *e*_*g*_ orbital occupancy at the octahedral sites in the spinel structure was analyzed. In the case of transition-metal catalysts, the electrocatalytic activity depends not only on the redox potential but also on the electronic configuration.^71^ Taking into account that the antibonding orbital *e*_*g*_ overlaps to a greater extent with the O 2p orbital (compared to the *t*_*2g*_ bonding orbital), it was found that the *e*_*g*_ orbital occupancy is one of the key descriptors of electrocatalytic activity.^45,72^

Wei et al. demonstrated that *e*_*g*_ occupancy regulates the bond strength between the catalyst and adsorbed/desorbed oxygen species, thereby influencing catalytic activity toward oxidation.^73^ In transition metals, the five *d* orbitals in the octahedral ligand field are split into a lower-energy triplet, *t_2g_*, and an upper *e*_*g*_ doublet. However, their ionic states are often not well-defined due to the Jahn–Teller distortion.

According to Waskowska et al., in the basic spinel CuMn_2_O_4_, the cation configuration was determined as Cu^+^_0.2_Mn^2+^_0.8_[Cu^2+^_0.8_Mn^3+^_0.2_Mn^4+^_1.0_]­O_4_. The electronic configuration of the *d* ^*4*^ orbital of Mn^3+^ is described as *(t*_*2*_^*3*^ *)­e*_*g*_^*1*^ and for Cu^2+^ with *d* ^*9*^ configuration, it is *(t*_*2g*_^*6*^ *)­(e*_*g*_^*2*^ *)­e*_*g*_^*1*^, where the configurations in parentheses denote paired electron spins.^72^

Substitution of manganese with other 3*d* metals (e.g., Ni) leads to the disappearance of the phase-transition characteristic of stoichiometric spinel. As previously demonstrated, nickel doping changes the Mn^3+^/Mn^4+^ ratio. The electron distribution of the Mn^4+^ ion, with the *e*_*g*_ orbitals being empty, is described as (*t*_*2g*_^*3*^ *)­e*_*g*_^*0*^. These vacant orbitals can accommodate ions of similar ionic radii but with different electronic configurations, thereby modifying the physicochemical properties of the material. In the case of Ni^2+^, the d^8^ orbital is *(t*_*2g*_^*6*^ *)­e*_*g*_^*2*^.

Doping can induce charge redistribution within the asymmetric TM–O–TM unit (TM—transition metal). In the work of Y. Yan et al., both experimental and theoretical results confirm that electron transfer occurs from the O_2p_ orbital to the TM (Ni^2+^ and Mn^4+^) *e*_*g*_ orbital via superexchange interactions in the Mn^4+^–O^2–^–Ni^2+^ coordination unit.^74^ As a result of this charge redistribution, the internal electric field enhances the electron and ion transport properties of the doped material.

It can, therefore, be concluded that nickel doping affects the electronic structure of the spinel and the ratio of manganese cations Mn^3+^/Mn^4+^, resulting in improved electrocatalytic activity and more effective glucose detection.

## 4 Discussion

This study presents a comprehensive analysis of manganese–copper spinel oxides intended for use as electrochemical glucose sensors, covering both their structural characteristics and their electrochemical activity. Particular attention was paid to the role of nickel doping and its influence on glucose-sensing properties. The electrochemical results clearly indicate that the introduction of nickel into the structure of the material leads to a marked improvement in catalytic efficiency toward the glucose oxidation reaction.

Based on the obtained XAS results (both EXAFS K-edge *operando* and L-edge), electrochemical data, theoretical calculations, and relevant literature,^60,75^ the following electrochemical reaction equations can be considered:

1 $${\mathrm{CuMn}}_{2-x}{\mathrm{Ni}}_{x}O_{4}+{\mathrm{OH}}^{-}+H_{2}O\to \frac{2-x}{2}{\mathrm{MnO}}_{2}+\frac{2-x}{2}\mathrm{MnOOH}+\mathrm{CuOOH}+x\mathrm{NiOOH}+e^{-}$$

2 $${\mathrm{MnOOH}+\mathrm{OH}}^{-}\to {\mathrm{MnO}}_{2}+H_{2}{O+e}^{-}$$

3 $${\mathrm{MnO}}_{2}+\mathrm{glucose}\to \mathrm{MnOOH}+\mathrm{gluconolactone}$$

4 $$\mathrm{MnOOH}+\mathrm{glucose}\to \mathrm{Mn}{\left(\mathrm{OH}\right)}_{2}+\mathrm{gluconolactone}$$

5 $$\mathrm{CuOOH}+\mathrm{glucose}\to \mathrm{Cu}{\left(\mathrm{OH}\right)}_{2}+\mathrm{gluconolactone}$$

6 $$\mathrm{NiOOH}+\mathrm{glucose}\to \mathrm{Ni}{\left(\mathrm{OH}\right)}_{2}+\mathrm{gluconolactone}$$

In the electrolyte, upon applying voltage, hydrolysis occurs, leading to the formation of MnO_2_ and complexes of MnOOH, CuOOH, and NiOOH. Analysis of L_3_-edge spectra (Figure 4) reveals that a higher nickel content in the sample shifts the oxidation state of manganese toward +4. The increased presence of Mn (IV) results in a higher amount of MnO_2_ in the electrolyte, which leads to the occurrence of the eq 4 and to increased oxidation of glucose to gluconolactone. This may explain the improved properties of CuMn_1.1_Ni_0.9_O_4_ in glucose detection. Considering the applied measurement potential, XANES measurements indicate that the redox signals recorded electrochemically are dominated by processes occurring in the (hydroxylated) surface layer, where the Mn^3+^/Mn^4+^ pair is the electroactive center in the studied potential window. This may be due to the stabilization of Mn^4+^ by nickel doping. In addition, the NiO/Ni­(OOH)_2_ redox potential occurs at higher potentials (∼1.35 V vs RHE).^76^

Although nickel incorporation enhances the electrocatalytic performance, its effect is not expected to increase indefinitely. Excessive substitution of Mn by Ni may reduce the concentration of electroactive Mn^3+^/Mn^4+^ couples and alter the cation distribution within the spinel lattice, potentially limiting the charge-transfer kinetics. Therefore, the enhanced performance of CuMn_1.1_Ni_0.9_O_4_ is likely associated with an optimal balance between the electronic conductivity and the concentration of redox-active manganese sites.

Furthermore, analysis of the K-edge EXAFS R-space Fourier transform indicates that, in the case of the doped sample, copper and nickel undergo neither oxidation nor reduction upon the addition of glucose, although under typical conditions, glucose addition should lead to their reduction. It is assumed that, during *operando* analysis, the dominant process shown by eq 2 is evident, involving manganese reduction and revealing Mn as the most active element. The combination of *operando* K-edge EXAFS and L-edge XAS measurements provides direct evidence that the electrochemical response originates predominantly from manganese-centered redox processes. This finding allows for distinguishing the role of individual cations and reveals that nickel acts primarily as an electronic promoter rather than as a directly participating redox center.

Atomistic analysis reveals that nickel doping in CuMn_2‑*X*_Ni_*X*_O_4_ spinel materials significantly alters their electronic structures, crystal geometries, and adsorption properties. The upward shift of the Fermi level from −7.10 eV (CuMn_2_O_4_) to −7.64 eV (CuMn_1.5_Ni_0.5_O_4_) indicates enhanced electron availability for redox processes, which is crucial for catalytic and electrochemical applications. This shift, combined with an increased band gap near the Fermi level, suggests modified charge-transport properties and increased material stability.

Moreover, doping introduces structural distortions, as evidenced by changes in bond lengths: Mn–O decreases, while Cu–O increases. Adsorption density improves, decreasing the molecular footprint from 63 Å^2^ per molecule (CuMn_2_O_4_) to 35 Å^2^ per molecule (CuMn_1.25_Ni_0.75_O_4_) in a vacuum, and only slightly increasing the molecular footprint from 35 Å^2^ per molecule (CuMn_2_O_4_) to 40 Å^2^ per molecule (CuMn_1.25_Ni_0.75_O_4_).

Adsorption energies also increase, reaching −2.91 eV for CuMn_1.5_Ni_0.5_O_4_ in vacuum and −2.50 eV in water, indicating stronger molecule–surface interactions. Furthermore, nickel doping facilitates stronger binding between glucose oxygen atoms and surface copper or manganese atoms—interactions absent on undoped surfaces. The stronger adsorption of glucose molecules observed for Ni-containing surfaces suggests a higher probability of charge transfer between glucose and the active surface sites, thereby facilitating the oxidation reaction. Consequently, the enhanced adsorption strength and reduced molecular footprint may contribute to the increased sensitivity observed experimentally.

Theoretical analysis of the occupancy of *e_g_*/*t_2g_* orbitals shows that nickel substitution modifies the electronic structure of the spinel by altering the Mn^3+^/Mn^4+^ ratio and introducing Ni^2+^ cations with distinct *e_g_* orbital occupancy. This doping-induced change in electron distribution, supported by superexchange interactions between Mn^4+^–O^2–^–Ni^2+^, enhances charge transfer and strengthens the internal electric field. Nickel substitution modifies the electronic configuration and may bring the *e_g_* occupancy close to the optimal value, thereby enhancing adsorption and charge-transfer processes.^77^ As a result, the material exhibits improved electrocatalytic activity, contributing to more efficient glucose oxidation and detection.

Overall, the improved glucose-sensing performance of Ni-doped CuMn_2‑*x*_Ni_*x*_O_4_ cannot be attributed to a single factor. Instead, it results from the synergistic interplay between enhanced electronic structure and stabilization of Mn^4+^ species. *Operando* XAS measurements further demonstrate that manganese acts as the primary redox center, while nickel serves as an electronic promoter, providing a comprehensive mechanistic understanding of the role of nickel substitution in spinel-based glucose sensors.

## 5 Conclusions

In conclusion, nickel-substituted manganese–copper oxide spinels, synthesized via the EDTA–CA method, demonstrate high electrochemical activity for glucose detection. This study confirms the strong influence of nickel substitution on the electrocatalytic properties of the materials, revealing a clear correlation between the amount of nickel added and the improved glucose oxidation. The electrodes exhibit linear responses across the tested glucose concentration range, combined with high sensitivity.

Selectivity tests further validate the suitability of these materials, showing negligible interference from common compounds such as sucrose and fructose. *Operando* XAS analysis revealed that nickel doping increases the concentration of Mn^4+^ cations in the spinel structure, contributing to enhanced catalytic activity. These findings are supported by theoretical calculations, which confirm the positive effect of nickel on the material’s electronic structure and adsorption behavior.

Overall, the results demonstrate the promising potential of nickel-doped manganese–copper spinels for the development of efficient, selective, and sensitive glucose sensors, with broad applications in electrochemical sensing technology.

## Supplementary Material



## Notes

Additional data, photographs, and analysis (PDF)
