# Supplementary material for: Electronic Structure Engineering of Cu–Mn Spinel Oxides via Ni Substitution for Enhanced Electrocatalytic Glucose Oxidation
Source: J Phys Chem C Nanomater Interfaces. 2026 Jul 4;130(28):9748–60. doi: 10.1021/acs.jpcc.6c03237 (PMC13387513; doi:10.1021/acs.jpcc.6c03237)
Supplement: Supplementary file 1 [file jp6c03237_si_001.pdf]

SUPPLEMENTARY INFORMATION

Electronic Structure Engineering of Cu-Mn Spinel Oxides via Ni Substitution for Enhanced Electrocatalytic Glucose Oxidation

Katarzyna Ostrowska [a]***, Bartłomiej Lemieszek*** [a]***, Krystian Lankauf*** [a]***, Iga Szpunar*** [a,b]***, Alexey Maximenko*** [c]***, Bartłomiej Dec*** [d]***, Piotr Jasiński*** [a]***, Sebastian Molin*** [a]

[a] *Advanced Materials Center, Faculty of Electronics, Telecommunications and Informatics, Gdańsk University of Technology, ul. G. Narutowicza 11/12, 80-233 Gdańsk, Poland*

[b] *Wallenberg Initiative Materials Science for Sustainability, Department of Chemistry and Chemical Engineering, Chalmers University of Technology, Gothenburg 41296, Sweden*

[c] *National Synchrotron Radiation Centre SOLARIS, Jagiellonian University, Czerwone Maki 98, 30-392, Kraków, Poland*

[d] *Department of Optoelectronics, Faculty of Electronics, Telecommunications and Informatics, Gdańsk University of Technology, 11/12 Gabriela Narutowicza Street, Gdańsk 80-233, Poland*

1. EXPERIMENTAL SECTION

(a) **(b)**

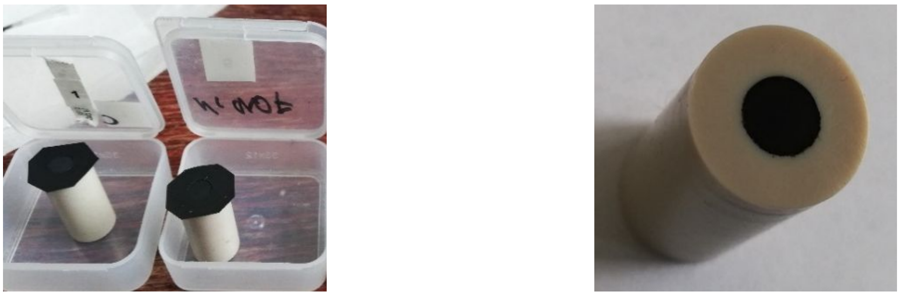

Fig. S1 *Example of the GCE modified electrode (a) electrode after the ink drop-casting with the tape mask and (b) electrode with the applied ink after mask removal.*

2. MATERIAL CHARACTERIZATION

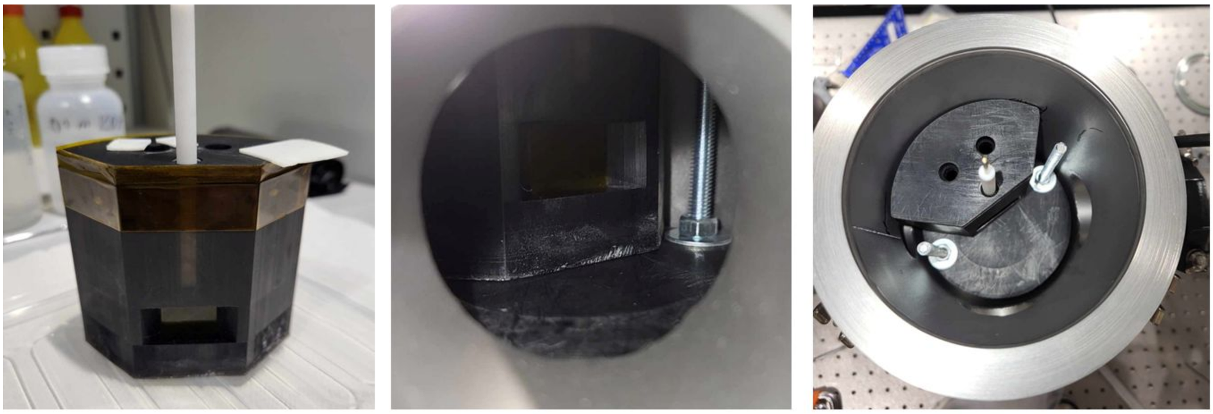

Fig. S2 *Operando* XAS electrochemical measurement setup

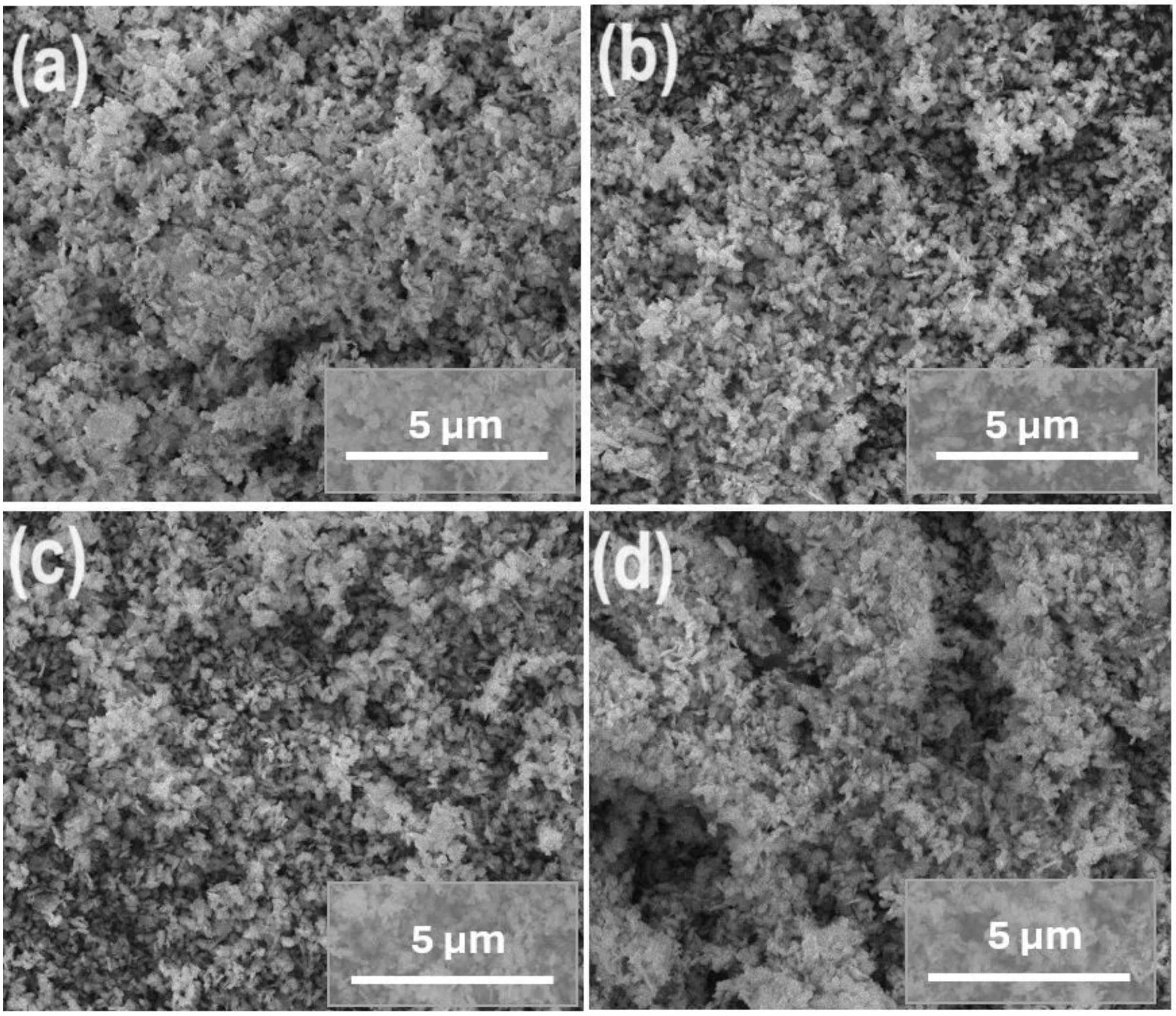

Fig. S3 SEM image (x10,000) of ball-milled (a) $CuMn_{1.1}Ni_{0.9}O_4$, (b) $CuMn_{1.4}Ni_{0.6}O_4$, (c) $CuMn_{1.7}Ni_{0.3}O_4$, (d) $CuMn_2O_4$ powder

Table S1 EDS analysis results

Material	Theoretical ratio Mn:Ni	EDS analysis result		
		Mn [at. %]	Ni [at. %]	Mn:Ni
**$CuMn_2O_4$**	---	100	---	---
**$CuMn_{1.7}Ni_{0.3}O_4$**	5.67	18.35	3.12	5.88
**$CuMn_{1.4}Ni_{0.6}O_4$**	2.33	12.71	5.26	2.41
**$CuMn_{1.1}Ni_{0.9}O_4$**	1.22	12.03	9.45	1.27

3. Electrochemical analysis

3.1 Error calculations

The nonlinearity error of each calibration curve was estimated according to the following equation:

$$\delta_{nl} = \frac{\Delta Y_{MAX}}{Y_{MAX} - Y_{MIN}} \cdot 100\% \qquad (1)$$

, where:

ΔY_{MAX} - maximum value of the absolute differences between the value determined on the basis of the equation of the straight line Y_{reg} (x) and the measurement results y = f (x), Y_{MAX} – maximum measured value, Y_{MIN} – minimum measured value.

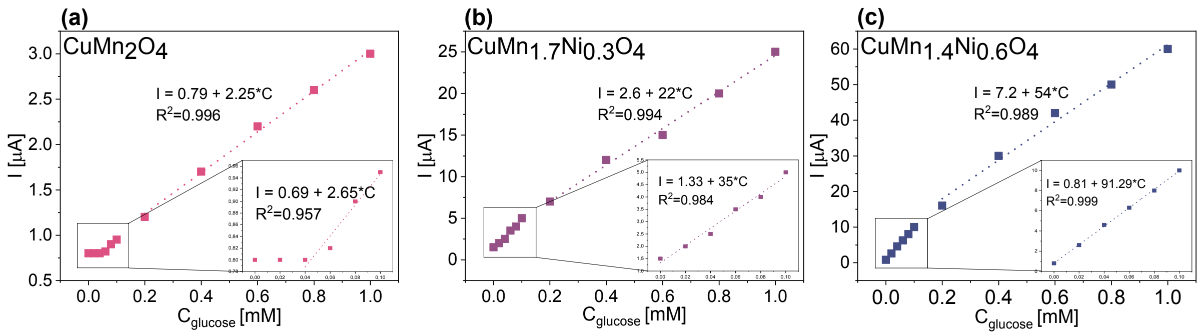

Fig. S4 Calibration curve with two linearity regions for (a) $CuMn_2O_4$, (b) $CuMn_{1.7}Ni_{0.3}O_4$, (c) $CuMn_{1.4}Ni_{0.6}O_4$.

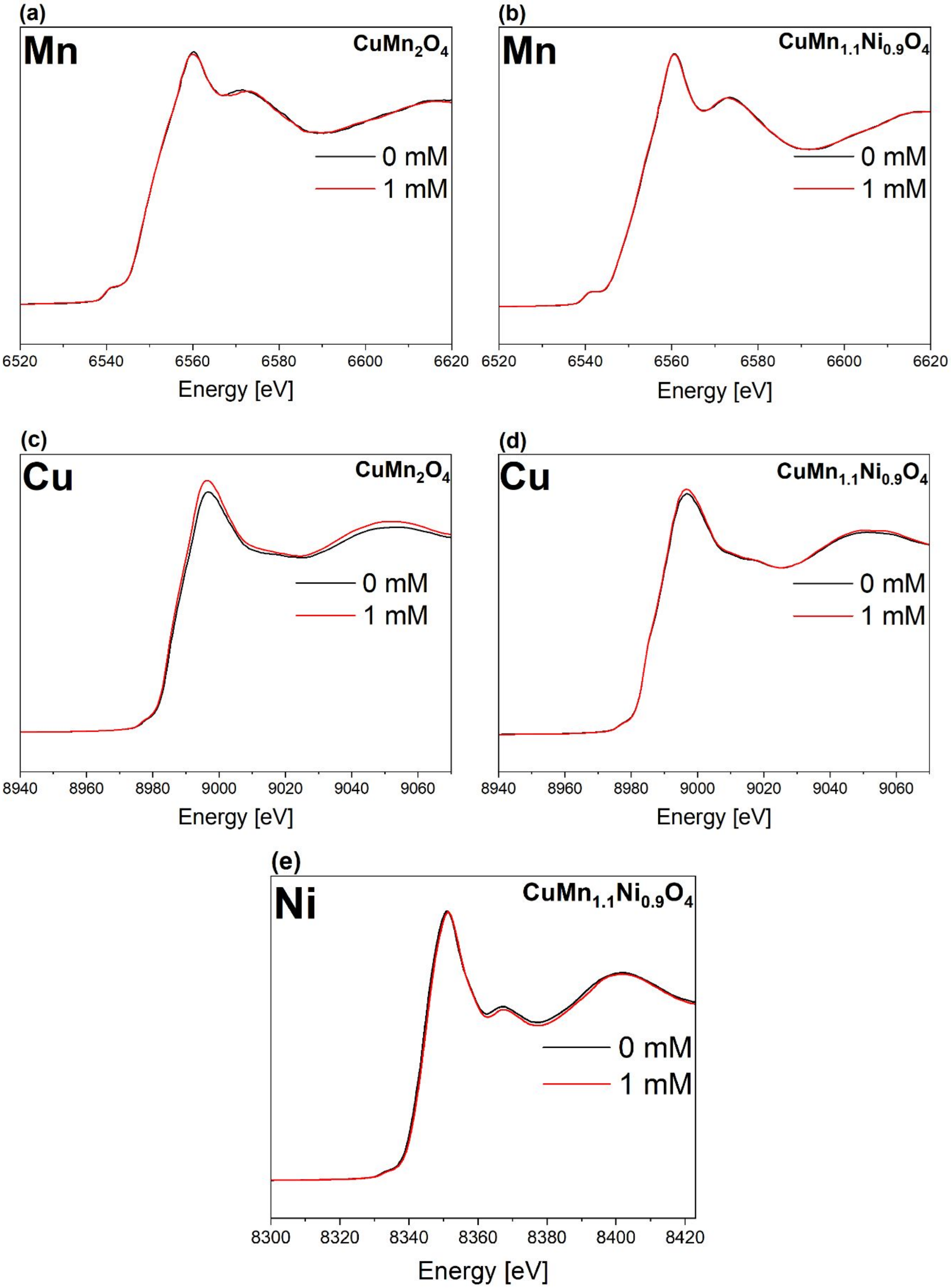

Fig. S5 XANES K-edge spectra of (a-b) Mn; (c-d) Cu; and (e) Ni.

Table S2 Fourier transform fit values for best fit.

$CuMn_{1.1}Ni_{0.9}O_4$	Shell	N	sigma^2	R	R-factor
**Mn**	Mn-O	4.52	0.0082	1.90	0.020
	Mn-Mn/Ni	4.37	0.0089	2.93	0.018
	Mn-Cu	4.32	0.0014	3.32	0.017
**Cu**	Cu-Mn/Ni	6.97	0.0101	3.43	0.08
	Cu-O	3.32	0.0036	1.93	0.06
	Cu-Cu	3.28	0.0130	3.75	0.11
**Ni**	Ni-Mn/Ni	4.60	0.0064	2.95	0.003
	Ni-O	5.42	0.0118	2.06	0.003
	Ni-Cu	3.22	0.0178	3.46	0.003

N - coordination number; R - interatomic distance; sigma^2- disorder parameter. All the fitting analysis were performed in the R space, ΔR = 1.3−4.1 and ΔK = 3−7.8.

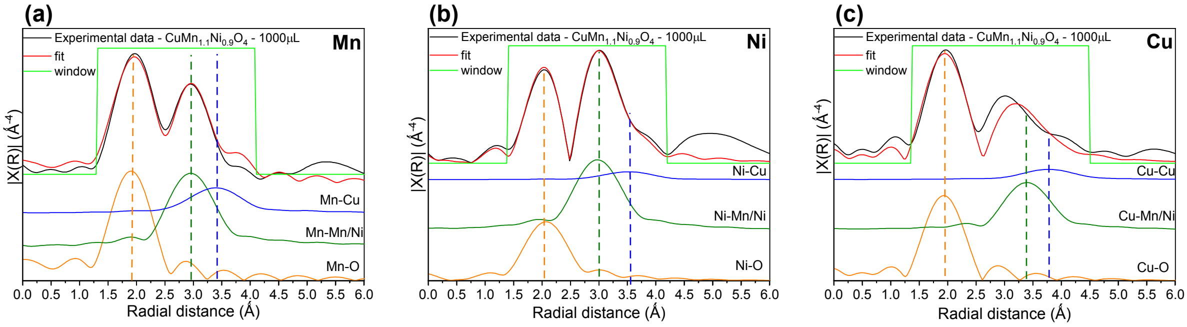

Fig. S6 Determined the vibration spectra |X(R)| (Å-4) in k-space with a fit for central atom of (a) Mn, (b) Ni, and (c) Cu.
